# Cholera, as It Appeared in Clarksville, Tennessee; with Reflections

**Published:** 1849-07

**Authors:** E. B. Haskins

**Affiliations:** Clarksville


### WESTERN JOURNAL

OF

### MEDICINE AND SURGERY.

---

JULY, 1849.

---

Art. I.—*Cholera, as it appeared in Clarksville, Tennessee; with Reflections.* By Dr. E. B. Haskins.

In my letter of the 10th of March, I refrained from offering any views of my own with regard to the pathology of Asiatic cholera, as well as from making any suggestions respecting the treatment of the disease, having never, at that time, seen a case professionally. Since that time, however, cholera has visited our town in quite a marked character, and having taken some share in its management myself, I proceed to give a somewhat detailed accout of its invasion and spread, type or character, the treatment employed, the results, etc. To which I will add some reflections, which I trust will not be unacceptable to the readers of the Journal.

The disease made its appearance here on the 16th of March, and disappeared about the 27th. During that time the physicians reported to the board of health (composed of Drs. Donoho, Cooper, and myself) sixty-six cases, and eighteen deaths.

The information given to the physicians, as a guide for them in making out their reports to the board of health, was, that no case would be received as a case of cholera, where the symptoms were simply those of diarrhea without the rice-water characteristic, and unattended with exhausting influences, vomiting or cramps; but that all cases of diarrhea, even if unaccompanied with puking or cramps, or both, *if the discharges have the rice-water characteristic, and be attended with progressive weakness and exhaustion*, would be received as cholera cases.

Notwithstanding the care exercised to procure correct statistics, it is possible, owing to the difficulty necessarily attending the diagnosis of a disease, the first, and most important stage of which simulates so nearly other disorders, that a few cases may have been reported which were not of epidemic origin; but it is believed that others were left out of the reports, having such origin, and therefore the number reported may be regarded as very near the truth.

In the invasion and spread of cholera over our town, numbering about three thousand inhabitants, we have been unable to detect anything resembling the invasion and spread of a contagious disease. In the very beginning of the epidemic, several individuals, residing in different portions of the town, and having had no communication with one another, were attacked about the same time; and during its whole continuance, the cases appeared to arise irrespective of any proximity to the sick or their residences, the nurses of the sick, and inmates of the same apartments, escaping in nearly all instances.

The disease having already prevailed for some time previously in both the cities of New Orleans and Nashville, and the steamboats daily landing passengers and merchandise upon our wharves, we were furnished with no facts upon which to found a conclusion as to whether it was brought up or down the river;* or what time intervened from the dissemination of the poison, to its manifestation in disease. One fact more should be stated, and that is, that no patient with cholera had been landed here, and that our first patients of cholera had seen no cases of that disease.

The disease prevailed principally among the negro and destitute white population, and but few women or children of any class were subjects of the malady. No white persons residing in the more cleanly portions of the town, and enjoying the ordinary comforts of life were attacked, except a few, who were predisposed to gastro-enteric irritation, and even they, without exception, recovered. Disturbances of the healthy action of the stomach and bowels prevailed very generally.

The weather, for several weeks prior to the invasion of the epidemic, had been cloudy and rainy, with sudden alternations of temperature, from warm to cold days, and *vice versa*. The atmosphere for most of the time was hazy.

*Symptoms and progress.* The first stage, or stage of serous evacuations, was ushered in by diarrhea, more or less violent, consisting, in the beginning, of feces dissolved in the intestinal serosity, giving the discharges, after the first two or three evacuations, the appearance of dirty soapsuds; which appearance, as the disease progressed, was gradually lost in that of the well-known

---

* Clarksville is situated on the Cumberland river, 65 miles below Nashville.

rice-water discharges. Sometimes vomiting and muscular spasms appeared very early in this stage; but generally they were later and announced the near approach of the second stage. Vomiting and cramping, however, were both often absent in this stage. In a large majority of the cases, the first stage was not a rapid one, the serous evacuations continuing from twelve to forty-eight, and, in one of my own cases, seventy-two hours, before the accession of the second stage forced the patients to take their beds and make known their condition. A few cases, however, were much more rapid, the first stage lasting only some six or eight hours. The pulse in this stage seldom underwent any perceptible alteration; the appetite was gradually lost, and the tongue was slightly coated with a white fur.

The second, or cold stage, or stage of depression, came on with feelings of more or less exhaustion; with restlessness, the patient often tossing violently from side to side; cool and shrunken surface, sometimes dry; but generally a dewy perspiration stood upon the face and breast, and later, when collapse announced the termination of this stage in death, the whole body was bathed in a cool sweat; features sharpened; eyes sunken, dull, and heavy; voice weak and husky; tongue cold, flabby, aud without moisture; frequent craving of cold drinks; pulse but little accelerated, depressed, and feeble, generally eighty or ninety, (I found it in one fatal case one hundred and twenty); urinary discharges slight, or entirely suppressed; serous evacuations sometimes continued unabated, but for the most part they were mitigated and sometimes entirely controlled; retching generally continued through this stage; cramps were aggravated until near its termination, when, in most cases, they ceased to give annoyance.

In those who died in this stage, the exhaustion and sinking gradually increased, when collapse supervened, carrying the patient off in a state of quiet insensibility. In those who survived the second stage, after the successful arrest of the serous evacuations, the subsidence of the spasms, etc., the third, or febrile stage, or stage of reaction followed. The pulse gradually rose in fulness, and slightly increased in frequency, beating from ninety to a hundred in a minute; warmth was gradually diffused over the skin; the tongue became lively and moist; craving for cold water grew less; and in those cases that recovered, the dejections were freely mixed with bile; the kidneys resumed their function; the appetite returned, and after a few days of moderate fever, health was restored. But in those that proved fatal, the kidneys never resumed their proper action:* thirst continued more or less to harrass the sufferer, and in twelve or twenty-four, and sometimes as late as forty-eight hours or upwards, after reaction was established, a drowsiness supervened with more or less delirium, but without any complaints of the head; the conjunctival membrane became red, and the pupils of the eye permanently contracted; the respiration reduced in frequency, (in one case to eight in the minute,) as the stupor deepeend; the bowels were discharged involuntarily; tracheal rattle set in, and the patient died in a state of profound coma, from the second to the fourth day of reaction.

---

* Notwithstanding the dissections in this and in other countries too clearly prove that the fatal stupor which supervenes in this stage, is dependent upon inflammatory (?) congestion of the brain and its membranes, yet may not urea and the urates (after the arrest of the serous evacuations, which, perhaps, were conducting off, by this channel, the nitrogenous effete matters), have some agency in determining the burthen of morbid action to this organ?

*Post-mortem examinations.*—But two dissections were made. In one, by Dr. Cooper, of a patient who died in the third stage, with symptoms of pneumonia, the right lung was found hepatized at its base; no other organs were examined. The other dissection was made by Dr. Finley and myself, in the presence of Drs. Cooper, Donoho, and McDaniel. This was a well-marked case of fatal coma in the third stage. The membranes of the brain presented extensive and extreme venous and capillary engorgement, with about four ounces of fluid in the base of the cranium; the ventricles contained no fluid;* no other cavity was opened.

*Treatment.*—The basis of our treatment consisted in the use of calomel, opium, and quinine, with such adjuncts as each practitioner selected to suit his own peculiar views. I will only, however, give a detailed account of the treatment pursued by myself and partner, Dr. W. M. Finley.

In the first stage, we relied mainly upon the horizontal posture, and the internal exhibition of calomel and opium—two grains of the former and one of the latter, made into a pill and given every half hour, or hour, until the evacuations were controlled, or the specific action of the opium was induced, with the free use of cold or warm diluents, according to the desire of the patient. When the evacuations were very frequent or abundant, from two to four of the calomel and opium pills were given for the first dose, and repeated, one at a time, as first mentioned. When this treatment was not sufficiently prompt in its action to give assurance of the prevention of the second or cold stage, we gave, along with the cal-

* It may be proper to state that between the hemispheres of the brain a well organized ossific deposit was found on the inner surface of the dura mater, twice the size of the thumb nail.

omel and opium pills, quinine in five grain doses, or sulph. quinine and pulv. camphor *āā* five grains. Ice seemed to control the vomiting better than anything else. To allay the spasms we used chloroform as follows:

Chloroform, ʒj.

Mucilage of gum arabic, ʒvij. Mix.

A teaspoonful in an ounce or more of ice water every fifteen or twenty minutes, until the spasms are allayed, or intoxication induced. This treatment, when early applied, scarcely failed in a single instance to prevent the accession of the cold stage, and cure the patient in a short time.

In the second stage, besides such remedies as have already been mentioned for the arrest of serous evacuations, relief of cramps, etc., we used warmth to the surface by means of hot bricks, pillows, and bottles filled with hot water, placed around the body and about the extremities of the patient.

In one case of collapse we tried the cold dash with apparent benefit; the patient who, before, was throwing himself about from side to side, with a cold damp skin, and nearly pulseless arm, was calmed; the pulse rose, and warmth partially returned to the surface; but we were unable to maintain the advantage, and he finally died.

In the third stage, to prevent and relieve the inflammatory congestion of the brain, we relied mainly upon topical bleeding from the nape of the neck, and between the shoulders, followed by blisters, hot foot-baths of mustard and salt, and cold applications to the head. We never, however, relieved a case, after the supervention of this condition.

REFLECTIONS. 1. *The Statistics of Cholera.*—The point in the statistics of cholera to which I wish particularly to invite attention at this time, as not the least embarrassing, is the frequent neglect of indicating, in the various reports, the group of symptoms recognized as constituting the disease *malignant cholera.* When, in the concatenation of morbid developments, from the simplest form of the first stage, to the collapse of the second stage, on the one hand, and reaction and visceral hyperemia of the third stage, on the other, does cholera commence? Some writers, it is true, speak of the diarrhea as the first stage of cholera; but others call it the premonatory symptom. Hence it is obvious, that the disease reported as *cholera* will show a very marked difference in its rate of mortality, according as cases recovering in the first stage be reported as such or not.

That the stage of serous evacuations is a part of the diseased action originating from the impression of the same poison that causes the second and third stages, perhaps no one will question. Indeed, being the nearest phenomenon to the cause, it is the most proximate effect; the other two stages being further removed are more remote effects; besides, this stage constitutes the most essential characteristic of the disease, since it is less frequently absent, if absent at all, than any other abnormal phenomenon; and hence the claims of science as well as public policy demand that cases recovering from this stage, if *clearly of epidemic origin*, should be embraced in the statistics of cholera. By this means alone can we be made acquainted with its actual and relative ravages, mortality, etc., and the actual and relative proportion of cases dying and recovering in the different stages.

In this way will the first and most important, but less alarming stage, be given that prominence which is necessary to its early and prompt attention; and by the exhibition of the true rate of mortality, and the success of medicine we shall allay the fears of the affrighted, as well as inspire confidence in medical treatment; both of which ends are so requisite to the successful management of the disease.

2. *Cause and Prevention.*—I have already offered for the consideration of the readers of the Journal, a theory of the propagation and spread of cholera, which in my humble judgment merits some consideration. (See May number.)

Acting upon the probability of the truth of this theory, the indications of prevention would be, first, to remove all sources of organic effluvia practicable; secondly, to prevent, as far as possible, the introduction of the cholera ferment; and thirdly, to prevent or arrest the process of effluvial fermentation.

The first indication will, of course, be best met by cleaning up and removing, or destroying by combustion, all organic matter in a state of decomposition, or a state subject to decomposition, from streets, alleys, ditches, sewers, cellars, pools, and other depositories of such remains; and by removing from closets and sleeping apartments, dirty clothing, and other accumulations of animal excrementitious matters. The second indication will be fulfilled by quarantine regulations; and the third, by the liberal use of unslacked lime in damp places, lime white-washing of fences, cellar walls, the outside and inside of out-houses, and the trunks of trees, so as to present as extensive absorbing surface as possible; and the sprinkling over the floors of basement-rooms, privies, etc., with chloride of lime, or chloride of zinc. These means may the more earnestly be insisted upon, as they all tend, independently of their effects upon the poison of cholera, to the preservation of health.

3. *Nature and Treatment of Cholera.*—Because cholera is not successfully treated in its most aggravated form, or in its last and most fatal stages, it is generally believed that its pathology is less understood, if understood at all, than, perhaps, any other of the acute diseases; and that therefore no reliable treatment can be deduced from its nature, nor from the experience and observation of the profession.

Without arrogating to myself the ability to offer a theory of the pathology of this disease, it may not be uninteresting to make some inquiries as to what we do know of cholera, in comparison with our knowledge of some other more familiar acute diseases, the treatment of which is, to a considerable extent, governed by professional authority. In the first place, it may be affirmed that notwithstanding post-mortem dissections seldom fail to disclose certain anatomical changes in some of the viscera of the head, thorax, and abdomen; yet it is scarcely contended by any one that cholera is essentially a local disease. That the local determinations are subsequent to a general constitutional disturbance, is a doctrine pretty generally admitted, I believe. So far then, the nature of cholera is no more obscure than that of bilious fever, pneumonia, rheumatism, and many other acute febrile diseases. Again, that this general constitutional disturbance has its principal location either in the vascular or nervous system, or partly in both, deranging, as a consequence of functional dependance, the secretory, excretory, and nutritive functions, would scarcely, I presume, be denied by any one; and in this also, cholera is no less understood than bilious fever, yellow fever, etc. And again, that this general constitutional disturbance, with subsequent derangement of the functions of the peripheral vascular system, produces* in

* Perhaps it would have less the appearance of hypothesis to say that those manifestations succeed one another, rather than that one produces the other, as facts are mainly considered.

rapid succession, certain local determinations made manifest by their peculiar signs and symptoms, is also a matter of almost universal observation, and in this too cholera is as well understood as many other diseases more familiarly known to the profession.

Of the essential nature of cholera, the nature and seat of the first link in the chain of morbid phenomena, and the nature and seat of the various successive links, and their relationship as cause and effect, ingenious and plausible theories may be framed; but nothing satisfactory is known, or probably can be known, in the present imperfect state of physiological and pathological science; nor can more be said of any other disease.

On the accession of any new disease, presenting a group of morbid phenomena, common to other, and familiar diseases, the prudent and enlightened practitioner would, undoubtedly, after a careful analysis of the symptoms, prescribe such remedies as the analogy of the morbid phenomena present, to those of other familiar diseases, would seem to indicate. This has been done in the case of cholera, and it is now my purpose to inquire whether or not the profession have been deceived in the result. In the first stage, or that of serous evacuations from the stomach and bowels, opiates, astringents, and by many calomel, have been used, from their acknowledged powers over such phenomena in other diseases. Have these remedies met the reasonable expectations of the profession? I think they have. There is, perhaps, not an opinion more common upon any subject of therapeutics, than that the first stage of cholera, if early attended to, can be easily controlled by some form or combination of these remedies. It would be unreasonable to expect that a disease, which if left without treatment, runs so rapidly and certainly a fatal course, should be relieved as promptly as diseases of a less fatal tendency.

But this is not the entire view of the subject. It becomes a question with the medical practitioner of no slight importance, whether in the sudden arrest of this discharge nature may not be defeated in her efforts to free the circulating mass of a morbific matter, and though the immediately exhausting effects of the loss of the vital fluids may be averted, yet other, and perhaps equally fatal results, may supervene from its retention in the circulation. Nor does experience allay such fears; for often, after the arrest of the serous evacuations, and the reaction of the system from the depressing stage, another fearful pathological development displays itself in drowsiness, delirium, and, too often, fatal coma. That the evacuations should be lessened in frequency, and amount, is clearly manifest; for if allowed to flow unchecked, they most certainly prove fatal in a short time, as the mortality amongst the careless and ignorant too sadly testifies.

Then it seems to me that the point of greatest practical importance consists in so directing our remedial agents, that, whilst we arrest and control the too copious flow from the stomach and bowels, we open up some other less exhausting channel for the elemination of deleterious matters; and calomel, from its unquestionable powers in exciting the function of secretion, would seem to offer, upon rational grounds, the surest prospect of meeting the indications required. By exciting the action of the glandular structures, the current of morbid fluxion is directed from the gastro-intestinal mucous surface, and the vital organs are at the same time protected against the retention of a fatal poison. Another reason why calomel should form a part of the treatment of the first stage of this disease is, its known resolvent powers over inflammatory action, and particularly of the brain and its membranes;* and hence the propriety of its timely administration to meet, as well as avert, the exigencies of the stage of reaction. Nor does experience contravene these views of the efficacy of mercury in cholera, but on the contrary greatly strengthens them; for although its use is opposed by some of high authority, there is no one remedy, opium, perhaps, excepted, which has a deeper hold upon the confidence of the profession, in the treatment of cholera, than calomel.

In the second, or cold stage, analogy, as well as rational considerations would justify the use of external warmth, diffusible stimuli,† etc.; and notwithstanding the results have not been as satisfactory as desired, yet they still have the confidence of the profession. This is the depressed stage of a most fearful malady, and it is scarcely to be expected that remedies would control it as readily as the cold stage of diseases not considered grave.

The stage of reaction has received less attention from the profession than the two preceding stages. Some have failed to notice it, owing to its absence in some localities and seasons, or else to its having been overlooked, or regarded as an accidental development. From its character, however, as displayed in the epidemic of this place, as well as from the testimony of those whose opportunities for observation have been best, it may be remarked that when unaccompanied by local determinations, but little treatment is required. But owing often to the insidious invasion of local disease of an almost certainly fatal termination—often making its appearance

---

*Notwithstanding dissections have not conclusively established the presence of inflammation in the cranial contents, yet the redness of the conjunctiva, and contracted state of the pupils, together with the post-mortem appearances, render such a conclusion at least probable.

† Brandy and alcoholic tinctures, however, were hurtful in our hands.

when least expected—of course the greatest vigilance is requisite to conduct the patient through this stage.—When the state of the circulation would seem to justify it, moderate bleeding, general or local; warm stimulating pediluvia; cool applications to the head; mercury in small doses; febrifuge drinks; and the prohibition of every thing calculated to excite the brain, as conversation, noise, light, etc.; and when symptoms of local disease arise, topical bleeding, blisters, etc., would appear, both from the analogy of symptoms, and the observation and and experience of the profession, to constitute the most approved and reliable treatment.

Since writing the above, my friend and neighbor, Dr. N. L. Thomas, a physician of very accurate observation, has kindly furnished me with the details of an interesting case of cholera which recovered after the accession of delirium and stupor in the stage of reaction; and although a single case will not establish the efficacy of any treatment, yet, from the happy termination of the case, the treatment certainly deserves further trial. My space will only allow of a quotation from his interesting letter, referring to this particular condition. Dr. Thomas says: "About three or four o'clock, in the afternoon of the 29th of March, 1849, my patient, a wagoner, commenced driving his teams; the delirium increased till it ran into a profound stupor, in which state I found him this morning (30th). His pulse about ninety, full and strong; respiration only six in the minute; pupils contracted and immovable; no stools. Took about a pint of blood from the arm; gave ten grains of calomel, and applied cold to the head. Soon after, commenced giving fifteen grains of carb. potash every two hours, day and night. In the afternoon applied cups to the back of the neck, which bled freely: about night shaved his head and applied a blister plaster all over it." On the first day of April, the stupor was very nearly gone, respiration twelve a minute. The carb. potash was now discontinued, and a pill composed of one grain of calomel and two of quinine was given three times daily. He gradually recovered.

"I was induced to use the carbonate of potash," says Dr. Thomas, "from seeing it recommended in cerebro-spinal meningitis by Dr. Ames, of Mobile, Alabama. It is sometimes used in jaundice, and is supposed to promote the biliary secretion. It appeared to me to act in this case as a sudorific and diuretic, which I omitted to mention."

Clarksville, May 15, 1849.

